# Extrahippocampal Radiomics Analysis Can Potentially Identify Laterality in Patients With MRI-Negative Temporal Lobe Epilepsy

**DOI:** 10.3389/fneur.2021.706576

**Published:** 2021-08-04

**Authors:** E-Nae Cheong, Ji Eun Park, Da Eun Jung, Woo Hyun Shim

**Affiliations:** ^1^Department of Medical Science and Asan Medical Institute of Convergence Science and Technology, Asan Medical Center, University of Ulsan College of Medicine, Seoul, South Korea; ^2^Department of Radiology and Research Institute of Radiology, University of Ulsan College of Medicine, Asan Medical Center, Seoul, South Korea; ^3^Department of Pediatrics, Ajou University School of Medicine, Suwon, South Korea

**Keywords:** temporal lobe epilepsy, radiomics, laterality, extrahippocampal, magnetic resonance imaging

## Abstract

**Objective:** The objective of the study was to investigate whether radiomics features of extrahippocampal regions differ between patients with epilepsy and healthy controls, and whether any differences can identify patients with magnetic resonance imaging (MRI)-negative temporal lobe epilepsy (TLE).

**Methods:** Data from 36 patients with hippocampal sclerosis (HS) and 50 healthy controls were used to construct a radiomics model. A total of 1,618 radiomics features from the affected hippocampal and extrahippocampal regions were compared with features from healthy controls and the unaffected side of patients. Using a stepwise selection method with a univariate *t*-test and elastic net penalization, significant predictors for identifying TLE were separately selected for the hippocampus (H+) and extrahippocampal region (H–). Each model was independently validated with an internal set of MRI-negative adult TLE patients (*n* = 22) and pediatric validation cohort with MRI-negative TLE (*n* = 20) from another tertiary center; diagnostic performance was calculated using area under the curve (AUC) of the receiver-operating-characteristic curve analysis.

**Results:** Forty-eight significant H+ radiomic features and 99 significant H– radiomic features were selected from the affected side of patients and used to create a hippocampus model and an extrahippocampal model, respectively. Texture features were the most frequently selected feature. Training set showed slightly higher accuracy between hippocampal (AUC = 0.99) and extrahippocampal model (AUC = 0.97). In the internal validation and external validation sets, the extrahippocampal model (AUC = 0.80 and 0.92, respectively) showed higher diagnostic performance for identifying the affected side of patients than the hippocampus model (AUC = 0.67 and 0.69).

**Significance:** Radiomics revealed extrahippocampal abnormality in the affected side of patients with TLE and could potentially help to identify MRI-negative TLE.

**Classification of Evidence:** Class IV Criteria for Rating Diagnostic Accuracy Studies.

## Key Points

- In patients with temporal lobe epilepsy (TLE), significant radiomic features were found in both hippocampus and extrahippocampal regions.
- For MRI-negative TLE, extrahippocampal model showed higher diagnostic performance for identifying the affected side than the hippocampus model.
- This proof-of-concept study demonstrates potential utility of radiomic features in extrahippocampal region, and further studies are needed to be conducted for actual clinical performance.

## Introduction

Identification of the lateralization of temporal lobe epilepsy (TLE) is important for presurgical workup, and precise localization of the epileptogenic focus is associated with a good surgical outcome. However, up to 30% of TLE cases appear normal (“non-lesional” or negative) without hippocampal sclerosis (HS) on 3-T magnetic resonance imaging (MRI) (1). Metabolic imaging using [^18^F]fluorodeoxyglucose-positron emission tomography (FDG-PET) showed that hypometabolism is not restricted to the mesial temporal lobe and that two-thirds of patients have a hypometabolism pattern in the adjacent lateral and posterior temporal lobe (2, 3). Such MRI-negative but PET-positive TLE is a surgically remediable syndrome (4–7) that primarily involves the lateral temporal neocortex rather than mesial lobe structures. Additionally, radiation exposure exists in PET and may not be appropriate for patients especially in children, due to the potential for long-term toxicity. This opens up the possibility that identification of extratemporal abnormalities could predict laterality in patients with MRI-negative TLE. Use of a non-invasive and no radiation-based MRI imaging tool for radiomics will be beneficial for pediatric patients, who will benefit from early management and monitoring.

While MRI studies performing quantitative analysis have shown cortical and subcortical volume atrophy (8), such atrophy occurs in end-stage disease, and its use for timely diagnosis of TLE may be limited. The recently developed radiomic approach involves extracting quantitative high-throughput data and can provide insight into intraregional heterogeneity and reflect the spatial complexity of a disease (9, 10). Although radiomic studies in TLE have shown feasibility in diagnosing hippocampal sclerosis and TLE (2, 11), they have not included extrahippocampal analysis nor have separated patients with MRI-negative epilepsy. A recent MR spectroscopy study demonstrated heterogeneous abnormalities in N-acetyl aspartate (NAA)/choline metabolism in the extrahippocampal region (12) in both MRI-positive and MRI-negative patients with TLE; thus, extrahippocampal radiomic analysis may provide insight into the heterogeneous etiology of TLE.

We hypothesized that the radiomic features of extrahippocampal regions would provide useful information in patients with hippocampal sclerosis and those with MRI-negative TLE, as remote extrahippocampal regions are anatomically connected with mesial temporal structures. We, therefore, investigated whether the extrahippocampal radiomic features of patients with TLE differed between the unaffected and affected side, as well as whether they differed from those of healthy controls, in order to evaluate whether they could provide useful information for determining the lateralization of epilepsy.

## Materials and Methods

### Study Participants

This retrospective study was approved by the Institutional Review Board of Asan Medical Center, and the requirement for informed consent was waived. A search of the database of the Department of Radiology at our tertiary center was performed for January 2019 to October 2020, and 421 consecutive patients with epilepsy who underwent an MRI protocol were identified; among them, patients without an EEG examination (*n* = 103), patients with an unclear epilepsy diagnosis or classification (*n* = 98), and patients with a type of epilepsy other than temporal lobe epilepsy (*n* = 161) were excluded.

Three groups of study participants were included. The first group consisted of patients diagnosed with focal TLE with a structural abnormality of hippocampal sclerosis (*n* = 36), and the second group consisted of age- and sex-matched healthy controls (*n* = 50). The healthy normal controls were selected from the same institution and had no history of neurological diagnosis, no history of hypoxia, encephalitis, or traumatic brain injury, and underwent the same MRI protocol.

**Figure 1:**
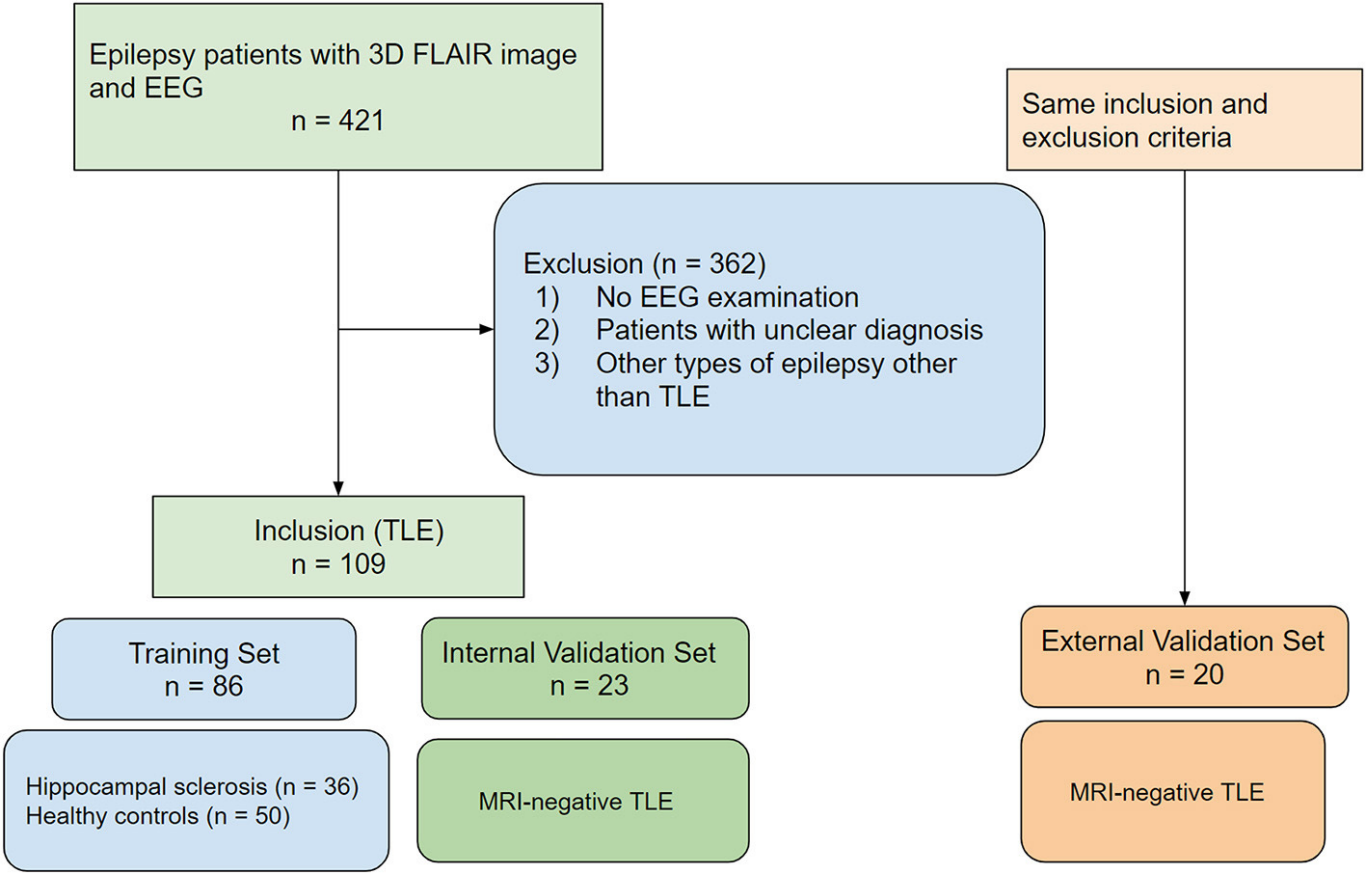
Flow diagram of patient inclusion and allocation to training and validation sets.

The first and second groups formed the training set used to develop the radiomic model. The third group consisted of patients with focal temporal lobe epilepsy without structural abnormality on MRI (MRI-negative TLE) and formed the validation sets for the radiomic model. These validation sets included internal validation consisting of adult patients with MRI-negative TLE (*n* = 23) and pediatric validation cohort with MRI-negative TLE from another tertiary hospital (*n* = 20). A flow diagram of the patient inclusion process is shown in Figure 1.

### Reference Standard for Temporal Lobe Epilepsy

The reference standard was the diagnosis of focal temporal lobe epilepsy, including both lesional and non-lesional epilepsy according to the International League Against Epilepsy (ILAE) classification of seizures and epilepsy (13). All patients had unilateral temporal interictal spikes or unilateral temporal lobe seizure onset on EEG. If both EEG and MRI findings showed involvement of the temporal lobe, the patient was diagnosed with TLE with structural abnormality.

### MRI Acquisition Protocol and Image Preprocessing

MRI was performed with a 3.0-T system with the following sequences: high-resolution anatomical three-dimensional (3D) T1-weighted and fluid-attenuated inversion recovery (FLAIR) volume imaging, axial and oblique coronal T2-weighted imaging, and gradient echo imaging. The 3D gradient-echo T1-weighted sequence was obtained using the following parameters: repetition time/echo time, 9.9/4.6 ms; flip angle, 8°; field of view, 220 × 220 mm; matrix, 224 × 224; and slice thickness, 1 mm with no gap. The 3D FLAIR sequence was obtained using the following parameters: repetition time/echo time, 4,800/320 ms; flip angle, 90°; field of view, 220 mm; matrix, 1,024 × 1,024; and slice thickness, 1 mm with no gap. The 3D FLAIR sequence, which had a high resolution and showed good signal differences, was used for the radiomics analysis.

### Segmentation and Radiomic Feature Extraction

An automatic brain segmentation and cortical parcellation were performed using Freesurfer (version 6.0; http://surfer.nmr.mgh.harvard.edu). The Desikian/Killiany atlas was used to define regions of interest (ROIs). From all ROIs in the temporal cortex of each hemisphere, the ROIs were combined to create four ROIs (right temporal, right hippocampal, left temporal, and left hippocampal ROI) in each patient. The segmentation process and region definition are described in detail in the Supplementary Materials.

All images were resampled to 1 × 1 × 1-mm resolution before the radiomic features were extracted. For FLAIR image data, signal intensity normalization was used to reduce variance in the signal intensity of the brain. We applied the hybrid white-stripe method (14) for intensity normalization using the ANTsR and WhiteStripe packages (15, 16) in R (version 4.0.3, R Core Team, Vienna, Austria). This method incorporates processes of the statistical principles of image normalization, preserving ranks among tissue and matching the intensity of tissues without upsetting the natural balance of tissue intensities (16). An automated process using Matlab (Matlab R2016a; Mathworks, Natrick, MA, USA) was used to extract the following three-radiomic feature groups: first-order features (*n* = 17), texture features (*n* = 162), and wavelet-transformed features (*n* = 1,432). Detailed information on the feature extraction is provided in Supplementary Table 1. The radiomic features we used adhered to the standards set by the Imaging Biomarker Standardization Initiative (IBSI) (17). The code for the feature extraction and analysis pipeline is available in an open repository [http://github.com/jieunp/radiogenomics~Radiomics-Code-AMC-Anew].

### Study Design and Statistical Analysis

#### Study Design

The radiomic features for predicting laterality were selected using (1) the *hippocampus* (H+), determined by comparing the affected-side hippocampus in patients (*n* = 36) with the unaffected-side hippocampus in patients and healthy controls [*n* = 86 (36 + 50)], and (2) the *extrahippocampal temporal region* (H–), determined by comparing the affected-side extrahippocampal region in patients (*n* = 36) with the unaffected-side extrahippocampal region in patients and healthy controls [*n* = 86 (36 + 50)]. The rationale for including hippocampal sclerosis were as follows: (1) hippocampal formation in the location of disease involvement in hippocampal sclerosis, which served as a reference model; (2) extrahippocampal locations are affected as extension of disease in patients with hippocampal sclerosis and would become a helpful reference model when we train the model in the ipsilateral side of hippocampal sclerosis; and (3) there were small number of patients of MRI-negative patients with TLE to be trained.

#### Statistical Analysis

**Figure 2:**
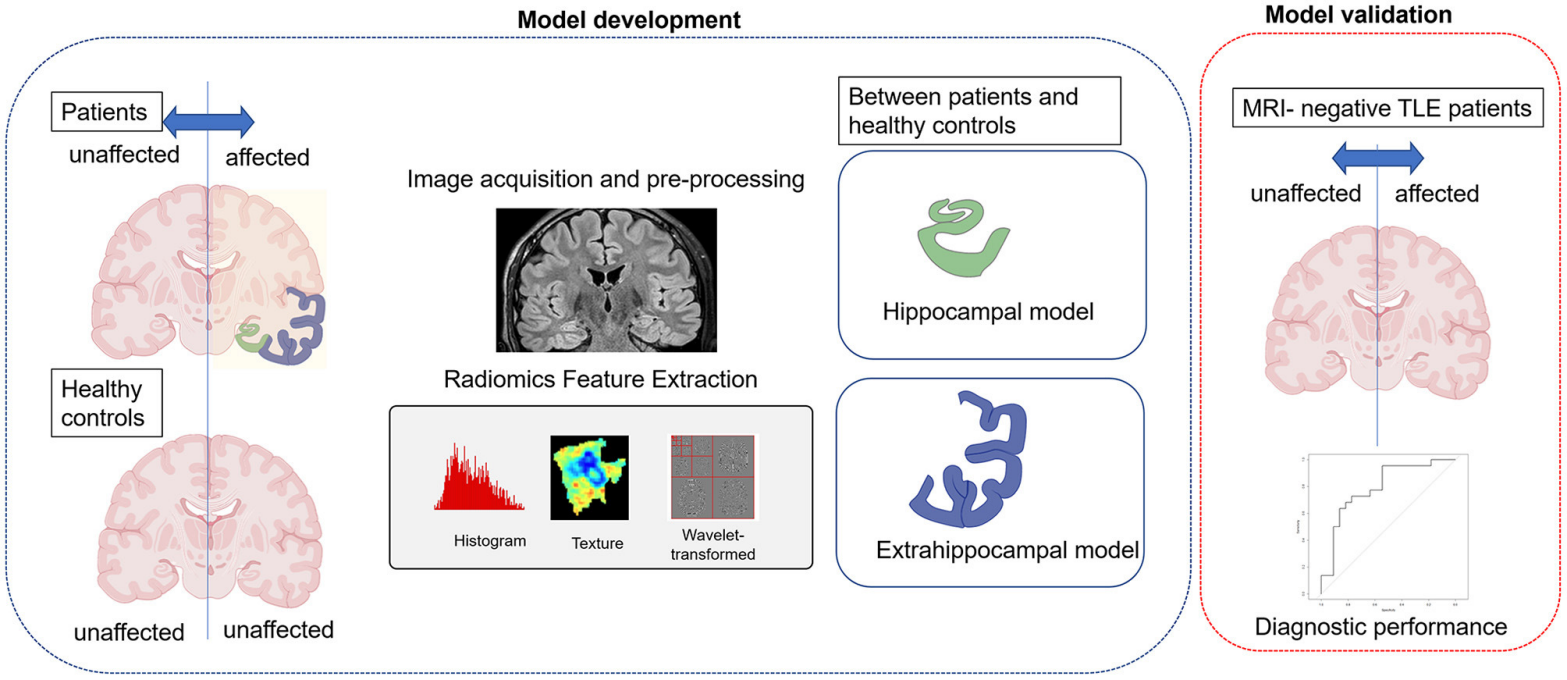
A schematic diagram of the study. First, hippocampal and extrahippocampal radiomic models were constructed using the training set and features showing significant differences between the affected and unaffected side. Second, independent validation was performed in patients with magnetic resonance imaging (MRI)-negative temporal lobe epilepsy (TLE) to evaluate the models' ability to diagnose laterality (the affected side).

The radiomic model construction involved two steps (Figure 2). The first step was to identify the significant radiomic features for predicting each condition using supervised learning (filtering). Student's *t*-tests were applied to all features for positivity for TLE diagnosis, with a *p*-value correction for multiple comparisons being performed using the false discovery rate with the Benjamini–Hochberg procedure. Then, the feature selection and modeling was performed by using logistic regression with elastic net regularization, a combination of the least absolute shrinkage and selection operator (LASSO) and ridge penalization method (18, 19). Two hyperparameters for elastic net were defined: α (range, 0–1), which determines the relative weights of LASSO (α = 1) and ridge (α = 0); and λ, a tuning parameter to determine the magnitude of penalization. The hyperparameters were optimized by using 10-fold cross validation based on mean squared error with the development data set divided nine-to-one using the “*glmnet*” package in R (version 4.0.3).

The diagnostic performance for predicting laterality was calculated using the area under the curve (AUC) of the receiver operating characteristic curve (ROC). The optimal thresholds for the AUCs were determined by maximizing the sum of the sensitivity and specificity values for predicting the diagnosis.

Student's *t*-test and Fisher's exact test were used to assess differences in the demographic data between the training and validation sets. All statistical analyses were performed using R software (version 4.0.3), with *p*-values <0.05 being deemed statistically significant.

## Results

### Study Participants

The baseline characteristics of the patients and healthy controls are shown in Table 1. The mean age of the patients with TLE in the training set was 25.5 years [standard deviation (SD), 9.0 years], and 66.7% were female. The affected hemisphere was the left side in 52.7% of the patients and the right side in 47.2%. The mean age of the healthy controls was 29.8 years (SD, 10.0 years) and 48.0% were female.

**Table 1:** Patient characteristics in the training and validation sets.

**Clinical characteristics**	**Training set**		**Internal validation set**	**External validation set**
	**Patients**	**Healthy controls**	**MRI-negative patients**	**MRI-negative patients**
	***n* = 36**	***n* = 50**	***n* = 22**	***n* = 20**
Age (years)	25.5 ± 9.0	29.8 ± 10.0	29.2 ± 8.6	15.9 ± 4.0
Sex (number of females)	24 (66.7%)	24 (48.0%)	12 (54.5%)	9 (45%)
Laterality				
Right	17 (47.2%)	–	10 (45.5%)	8 (40%)
Left	19 (52.7%)	–	12 (54.5%)	12 (60%)

*MRI, magnetic resonance imaging*.

### Significant Radiomic Features in the Hippocampus and Extrahippocampal Region

Within the patients, 684 features showed a significant difference between the affected and unaffected hippocampus according to univariate *t*-tests; the elastic net penalization method applied to these features showed 48 features to be predictive of the laterality of epilepsy (α = 0.9). The selected features are shown in Table 2.

**Table 2:** Selected radiomic features for predicting laterality after univariate filtering and penalization method.

**Feature type**	**Number of features**	**Filter**
**(A) Hippocampus (** ***n*** **=** **48)**		
First order	1	None
GLCM dist = 1/dist = 2/dist = 3	6	None
GLCM dist = 1/dist = 2/dist = 3	10	LLL
GLRLM	1	LLL
GLCM dist = 1/dist = 2/dist = 3	14	LLH
GLRLM	1	LLH
GLCM dist = 1/dist = 2/dist = 3	15	LHL
**Radiomic feature**	**Filter**	**Feature type**
**(B) Extrahippocampal region (** ***n*** **=** **99)**		
First order	4	None
GLCM dist = 1/dist = 2/dist = 3	8	None
GLRLM	4	None
GLCM dist = 1/dist = 2/dist = 3	21	LLL
First order	1	LLH
GLCM dist = 1/dist = 2/dist = 3	20	LLH
GLRLM	2	LLH
First Order	1	LHL
GLCM dist = 1/dist = 2/dist = 3	20	LHL
GLRLM	2	LHL
GLCM dist = 1/dist = 2/dist = 3	16	LHH

*FLAIR, fluid-attenuated inversion recovery; std, standard deviation; GLCM, gray-level co-occurrence matrix; GLRLM, gray-level run-length matrix; H, high-pass filter; L, low-pass filter*.

Eight hundred and fifteen features showed a significant difference between the affected extrahippocampal regions of the patients and the unaffected extrahippocampal regions of the healthy controls according to univariate *t*-tests; the elastic net penalization method showed 99 of these features to be predictive of the laterality of epilepsy (α = 0.35). The optimization process of the model is shown in Supplementary Table 2.

However, comparisons between the affected and unaffected extrahippocampal region within patients did not reveal any feature as being predictive of the laterality of epilepsy.

### Diagnostic Model for Prediction of Laterality

The hippocampal-based model for predicting laterality showed an AUC of 0.99 (95% CI, 0.97–1.00) when applied to the training set, whereas the extrahippocampal model showed an AUC of 0.97 (95% CI, 0.94–1.00).

To validate the models, they were tested on two separate validation sets, as summarized in Table 3. For the internal validation set, the hippocampal model showed an AUC of 0.67 (95% CI, 0.54–0.71), indicating poor performance. However, the extrahippocampal model had an AUC of 0.90 (95% CI, 0.86–0.93) for prediction of laterality.

**Table 3:** Diagnostic performance of the radiomic models in the training set and in patients with MRI-negative temporal lobe epilepsy.

	**Training set**	**Internal validation set**	**External pediatric validation set**
Model	MRI-positive TLE	MRI-negative TLE	MRI-negative TLE
Hippocampal model (H+)	0.99 (0.97–1.00)	0.67 (0.54–0.71)	0.69 (0.60–0.77)
Extrahippocampal model (H–)	0.97 (0.94–1.00)	0.80 (0.76–0.83)	0.92 (0.86–0.98)

To validate the true model performance, MRI-negative patients with TLE were recruited from another tertiary medical center. When applied to this external validation set, the hippocampal model showed an AUC of 0.69 (95% CI, 0.60–0.77), indicating poor performance, whereas the extrahippocampal model gave an AUC of 0.92 (95% CI, 0.86–0.98) for prediction of laterality, with a sensitivity of 92% and specificity of 96%.

## Discussion

In this study, we identified radiomic features measured in the hippocampal and extrahippocampal regions of the affected side of patients with TLE that significantly differed from those of the unaffected side and those of healthy controls. We then evaluated whether these features could predict laterality in MRI-negative patients with TLE. The hippocampal radiomic model was predictive of laterality in patients with hippocampal sclerosis but was not a successful predictive marker in MRI-negative patients. Alternatively, the radiomic features of the extrahippocampal region identified laterality in MRI-negative patients with TLE, which may be helpful in patients with clinically suspected TLE without discernable evidence of structural abnormalities.

Two types of TLE were distinguished on the basis of histopathological findings: (1) TLE with mesial temporal sclerosis, and (2) TLE with a normal-appearing hippocampus on MRI (MRI-negative TLE). The latter has become of great clinical interest, and recent studies have found that both structural and functional abnormalities exist beyond the hippocampus. The mechanism for MRI-negative TLE is yet to be elucidated, but the harmful effects of epileptic propagation through neural networks result in cell loss and gliosis in the extrahippocampal region (20) and microdysgenesis in the temporal neocortex (21). Twenty-nine radiomic features showing differences between the affected and unaffected extrahippocampal regions were selected for the extrahippocampal-based model, which was considerably more than the 11 radiomic features used for the hippocampus-based model. Our findings agree with previous studies that indicate that radiomic abnormalities in TLE extend beyond the hippocampus with extrahippocampal regions being affected.

Entropy was the most frequently selected feature in both hippocampal and extrahippocampal models among texture features. Entropy reflects the uniformity or randomness of the intensities in an image (22), and its identification in this study implies that voxel-level heterogeneity was significantly different between the affected and unaffected sides on FLAIR images. Gray matter loss has been reported to be asymmetric (23) and heterogeneous (12) in neuroimaging studies, and metabolic imaging demonstrated that NAA/Cho + creatinine (Cr) reduction in the extrahippocampal region (12) was heterogeneous, suggesting a heterogeneous etiology of metabolic abnormalities in patients with TLE. MRI-based radiomic analysis may provide additional value to volumetric analysis showing chronic atrophy, as it may provide an earlier biomarker, and also has value for demonstrating lateralized differences in heterogeneity. Additionally, radiomics results are along with the previous resting-state functional MRI study (24), which demonstrated that non-invasive prediction on MRI is feasible, and accurate lateralization can be advantageous for treating refractory TLE.

Interestingly, no extrahippocampal radiomic features showed within-patient differences between affected and unaffected sides. In an MR spectroscopy study (12), lateralizing information was not found in MRI-negative patients with TLE, with low NAA/Cho + Cr being observed in the ipsi- and contralateral hemispheres. It may come from rich afferent connections from extratemporal association cortices, as well as other areas (25) that serve as functional vulnerability in bilateral hemispheres. Although lateralization was not significant within patients, radiomic features showing differences between patients and healthy controls helped to lateralize MRI-negative TLE. The value of these features may be strengthened if they correlate with postsurgical (seizure-free) outcomes in MRI-negative TLE.

Our study is subject to the limitations of a retrospective design and has a relatively small sample size. Indeed, the external validation set has a particularly small population, and it is difficult to draw a strong conclusion from our results. A prospective outcome-based study on lateralization using radiomic features, and subsequent surgery with validation using clinical outcomes is needed. Furthermore, patients with TLE in our study were younger than those in previous reports (3, 11), and the external validation was based on a pediatric population. The etiology may differ between adults with TLE and pediatric patients with TLE; nevertheless, our results demonstrate the potential of radiomic analysis in both adult and pediatric populations. Therefore, results of this study need to be more focused in potential predictive value of radiomic in the extrahippocampal regions and “proof-of-concept” study rather than the performance itself.

In conclusion, radiomics revealed extrahippocampal abnormalities in the affected side of patients with TLE, and radiomic analysis could potentially help to identify MRI-negative temporal lobe epilepsy.

## Data Availability Statement

The raw data supporting the conclusions of this article will be made available by the authors, without undue reservation.

## Ethics Statement

The studies involving human participants were reviewed and approved by Institutional review board of Asan Medical Center. Written informed consent to participate in this study was provided by the participants' legal guardian/next of kin.

## Author Contributions

E-NC contributed to the writing of the manuscript and statistical analysis. JP contributed to the conceptual design and writing of the manuscript. DJ contributed to the editing of the manuscript, conceptual design, and project integrity. WS contributed to the conceptual feedback and software support. All authors reviewed the manuscript.

## Conflict of Interest

The authors declare that the research was conducted in the absence of any commercial or financial relationships that could be construed as a potential conflict of interest.

## Publisher's Note

All claims expressed in this article are solely those of the authors and do not necessarily represent those of their affiliated organizations, or those of the publisher, the editors and the reviewers. Any product that may be evaluated in this article, or claim that may be made by its manufacturer, is not guaranteed or endorsed by the publisher.

## Abbreviations

TLE: temporal lobe epilepsy
HS: hippocampal sclerosis
MRI: magnetic resonance imaging
PET: positive.
