# Supplementary material for: Extrahippocampal Radiomics Analysis Can Potentially Identify Laterality in Patients With MRI-Negative Temporal Lobe Epilepsy
Source: Front Neurol. 2021 Aug 4;12:706576. doi: 10.3389/fneur.2021.706576 (PMC8372821; doi:10.3389/fneur.2021.706576)
Supplement: Supplementary file 1 [file Table_1.docx]

**Supplementary Materials for**

**Extrahippocampal radiomics analysis can potentially identify laterality in patients with MRI-negative temporal lobe epilepsy**

*Definition of Regions of Interest*

The cerebral cortex was then parcellated into anatomical structures. Cortical thickness was computed by finding the shortest distance between a given point on the estimated pial surface and the gray matter/white matter boundary. Each thickness measurement of each vertex of the subjects' surface was mapped onto a standard spherical coordinate system. All temporal cortex regions of each hemisphere corresponding with those of the Desikian/Killiany atlas were combined to form a single ROI, e.g., the left- bankssts (1001), entorhinal (1006), fusiform gyrus (1007), lingual gyrus (1013), middle temporal gyrus (1132), planum temporale gyrus (1136), temporal pole (1143), lateral occipitotemporal sulcus (1163), medial occipitotemporal and lingual sulcus (1164), inferior temporal sulcus (1179), superior temporal sulcus (1180), and temporal transverse sulcus (1181) were combined to form a left temporal lobe ROI.

| **Label name** | **Label No.** | |
| --- | --- | --- |
|  | Left | Right |
| **Hippocampus** | 17 | 53 |
| **Temporal** |  |  |
| **Bankssts** | 1001 | 2001 |
| **Entorhinal** | 1006 | 2006 |
| **Fusiform gyrus** | 1007 | 2007 |
| **Lingual gyrus** | 1013 | 2013 |
| **Middle temporal gyrus** | 1132 | 2132 |
| **Planum temporale gyrus** | 1136 | 2136 |
| **Temporal pole** | 1143 | 2143 |
| **Lateral occipitotemporal sulcus** | 1163 | 2163 |
| **Medial occipitotemporal and Lingual sulcus** | 1164 | 2164 |
| **Inferior temporal sulcus** | 1179 | 2179 |
| **Superior temporal sulcus** | 1180 | 2180 |
| **Temporal transverse sulcus** | 1181 | 2181 |

**Supplementary Table 1. Radiomic features**

The first-order features described the histogram distribution of the entire tumor. The second-order features, including gray-level co-occurrence matrices and gray-level run-length matrices or texture features, captured the spatial mutual dependencies of the image voxels. The wavelet transformations extracted features from the wavelet-transformed space.

| **First order statistics**  $\boldsymbol{V}_{ROI}$ and $\boldsymbol{V}$ denote the intensity values of all voxels within $V(x,y,z)$ with $N$ voxels and the whole image, respectively. The mean and center gray values within $V(x,y,z)$ are $\overline{V}$ and $\boldsymbol{C}$, respectively | | | |
| --- | --- | --- | --- |
| **Number of voxels** | $\left\vert\boldsymbol{V}_{ROI} \right\vert$ | **Sum of intensities** | $\sum_{i}^{N} \boldsymbol{V}_{ROI}(i)$ |
| **Range** | $\text{max}\left( \boldsymbol{V}_{ROI} \right)-\text{min}\left( \boldsymbol{V}_{ROI} \right)$ | **Energy** | $N^{2}\sum_{i}^{N_{g}} {p\left( i \right)}^{2}$ |
| **Covered image intensity range** | $\frac{\max\boldsymbol{V}_{ROI}-\min\boldsymbol{V}_{ROI}}{\left( \max\boldsymbol{V}-\min\boldsymbol{V}+eps \right)}$ | **Entropy** | $-\sum_{i}^{N_{g}} \left[ p\left( i \right)*\log_{2} \left( p\left( i \right)+eps \right) \right]$ |
| **Maximum intensity value** | $\max\left( \boldsymbol{V}_{ROI} \right)$ | **Kurtosis** | $\frac{\sum_{i}^{N_{g}} \left[ p\left( i \right)*\left( \boldsymbol{C}\left( i \right)- \overline{V} \right)^{4} \right]}{\left( \sum_{i}^{N_{g}} \left[ p\left( i \right)*\left( \boldsymbol{C}\left( i \right)-\overline{V} \right) \right]+eps \right)^{2}}$ |
| **Mean intensity value** | $\frac{1}{N}\sum_{i}^{N} \boldsymbol{V}_{ROI}(i)$ | **Skewness** | $\frac{\sum_{i}^{N_{g}} \left[ p\left( i \right)*\left( \boldsymbol{C}\left( i \right)- \overline{V} \right)^{3} \right]}{\left( \sum_{i}^{N_{g}} \left[ p\left( i \right)*\left( \boldsymbol{C}\left( i \right)- \overline{V} \right)^{3} \right]+eps \right)^{\frac{3}{2}}}$ |
| **Median intensity value** | $\mathrm{med} \left( \boldsymbol{V}_{ROI} \right)$ | **Root means square** | $\sum_{i}^{N_{g}} \left[ p\left( i \right)*\boldsymbol{C}\left( i \right)^{2} \right]$ |
| **Minimum intensity value** | $\min\left( \boldsymbol{V}_{ROI} \right)$ | **Variance** | $\frac{1}{N-1}\sum_{i}^{N} \left( \boldsymbol{V}_{ROI}\left( i \right)- \overline{V} \right)^{2}$ |
| **Mean absolute deviation** | $\sum_{i}^{N_{g}} \left[ p\left( i \right)*(\boldsymbol{C}\left( i \right)- \overline{V}) \right]$ | **Standard deviation** | $\sqrt{\frac{1}{N-1}\sum_{i}^{N} \left( \boldsymbol{V}_{ROI}\left( i \right)- \overline{V} \right)^{2}}$ |
| **Uniformity** | $\sum_{i}^{N_{g}} p\left( i \right)^{2}$ |  |  |
| **GLCM features** | | | |
| Mean and standard deviation of the following for 13 directions and 3 distances  Frequently used feature quantities for each direction and distance are defined as follows:   - $\mu$ is the mean of $g\left( i,j \right)$ - $\sigma$ is the standard deviation of $g\left( i,j \right)$ - $g_{x}\left( i \right)$ is the marginal row probability of $g\left( i,j \right)$: $g_{x}\left( i \right)=\sum_{j}^{N_{g}} g\left( i,j \right)$, - $\mu_{x}$ is the mean of $g_{x}\left( i \right)$ - $\sigma_{x}$ is the standard deviation of $g_{x}\left( i \right)$ | | | |
| **autocorrelation** | $\sum_{i}^{N_{g}} \sum_{j}^{N_{g}} i*j*g(i,j)$ | **Haralick correlation** | $\frac{1}{\sigma_{x}}\sum_{i}^{N_{g}} \sum_{j}^{N_{g}} \left( i*j*g\left( i,j \right) \right)-\mu_{x}$ |
| **cluster prominence** | $\sum_{i}^{N_{g}} \sum_{j}^{N_{g}} \left( i+j-2\mu\right)^{4}*g\left( i,j \right)$ | **inverse difference** | $\sum_{i}^{N_{g}} \sum_{j}^{N_{g}} \frac{g\left( i,j \right)}{1+\left\vert i-j \right\vert}$ |
| **cluster shade** | $\sum_{i}^{N_{g}} \sum_{j}^{N_{g}} \left( i+j-2\mu\right)^{3}*g\left( i,j \right)$ | **inverse difference normalized** | $\frac{1}{N_{g}}\sum_{i}^{N_{g}} \sum_{j}^{N_{g}} \frac{g\left( i,j \right)}{1+\left\vert i-j \right\vert}$ |
| **cluster tendency** | $\sum_{i}^{N_{g}} \sum_{j}^{N_{g}} \left( i+j-2\mu\right)^{2}*g\left( i,j \right)$ | **inverse difference moment** | $\sum_{i}^{N_{g}} \sum_{j}^{N_{g}} \frac{g\left( i,j \right)}{1+\left( i-j \right)^{2}}$ |
| **contrast** | $\sum_{i}^{N_{g}} \sum_{j}^{N_{g}} \left( i-j \right)^{2}*g\left( i,j \right)$ | **inverse difference moment normalized** | $\frac{1}{{N_{g}}^{2}}\sum_{i}^{N_{g}} \sum_{j}^{N_{g}} \frac{g\left( i,j \right)}{1+\left( i-j \right)^{2}}$ |
| **correlation** | $\frac{1}{\sigma}\sum_{i}^{N_{g}} \sum_{j}^{N_{g}} \left( i-\mu\right)\left( j-\mu\right)*g\left( i,j \right)$ | **inverse variance** | $\sum_{i}^{N_{g}} \sum_{j}^{N_{g}} \frac{g\left( i,j \right)}{\left( i-j \right)^{2}}$ |
| **difference average** | $\sum_{k}^{N_{g}} k*g_{x-y}\left( k \right)$ | **maximum probability** | $max\left( g\left( i,j \right) \right)$ |
| **difference entropy** | $-\sum_{k}^{N_{g}} g_{x-y}\left( k \right)*{log}_{2}\left( g_{x-y}\left( k \right)+eps \right)$ | **sum average** | $\sum_{k}^{2N_{g}} i*g_{x+y}\left( k \right)$ |
| **difference variance** | $\sum_{k}^{N_{g}} \left( k-\bar{g_{x-y}} \right)^{2}*g_{x-y}\left( k \right)$ | **sum entropy** | $-\sum_{k}^{2N_{g}} g_{x+y}\left( k \right)*{log}_{2}\left( g_{x+y}\left( k \right)+eps \right)$ |
| **dissimilarity** | $\sum_{i}^{N_{g}} \sum_{j}^{N_{g}} \left\vert i-j \right\vert*g\left( i,j \right)$ | **sum variance** | $\sum_{k}^{2N_{g}} \left( k-\bar{g_{x+y}} \right)^{2}*g_{x+y}\left( k \right)$ |
| **energy** | $\sum_{i}^{N_{g}} \sum_{j}^{N_{g}} {g\left( i,j \right)}^{2}$ | **variance** | $\sum_{i}^{N_{g}} \sum_{j}^{N_{g}} \left( i-\mu\right)^{2}g\left( i,j \right)$ |
| **entropy** | $-\sum_{i}^{N_{g}} \sum_{j}^{N_{g}} g\left( i,j \right)*{log}_{2}\left( g\left( i,j \right)+eps \right)$ |  |  |
| GLRLM features | | | |
| Mean and standard deviation of the followings for 13 directions and 3 distances  $Q$ denotes the GLRLM of a quantized volume $V(x,y,z)$ with isotropic voxel size and $Q(i,j)$ represents the number of runs of gray level *i* with $j$ consecutive voxels in 1 of the 13 directions of $\alpha$. GLRLM is the size of $N_{g}\times N_{l}$ where $N_{g}$ describes the pre-defined number and $N_{l}$ represents the length of the longest run of quantized gray level sets in $V(x,y,z)$. $N_{p}$ is the number of voxels in $V(x,y,z)$. | | | |
| **number of runs** | $N_{run}$ | **low gray level run emphasis** | $\frac{1}{N_{run}}\sum_{i}^{N_{g}} \sum_{j}^{N_{l}} \frac{1}{i^{2}}*Q\left( i,j \right)$ |
| **gray level nonuniformity** | $\frac{1}{N_{run}}\sum_{i}^{N_{g}} \left( \sum_{j}^{N_{l}} Q\left( i,j \right) \right)^{2}$ | **run length nonuniformity** | $\frac{1}{N_{run}}\sum_{j}^{N_{l}} \left( \sum_{i}^{N_{g}} Q\left( i,j \right) \right)^{2}$ |
| **high gray level run emphasis** | $\frac{1}{N_{run}}\sum_{i}^{N_{g}} \sum_{j}^{N_{l}} i^{2}*Q\left( i,j \right)$ | **run percentage** | $\frac{N_{run}}{N_{p}}$ |
| **long run emphasis** | $\frac{1}{N_{run}}\sum_{i}^{N_{g}} \sum_{j}^{N_{l}} j^{2}*Q\left( i,j \right)$ | **short run emphasis** | $\frac{1}{N_{run}}\sum_{i}^{N_{g}} \sum_{j}^{N_{l}} \frac{1}{j^{2}}*Q\left( i,j \right)$ |
| **long run high gray level emphasis** | $\frac{1}{N_{run}}\sum_{i}^{N_{g}} \sum_{j}^{N_{l}} {i^{2}*j}^{2}*Q\left( i,j \right)$ | **short run high gray level emphasis** | $\frac{1}{N_{run}}\sum_{i}^{N_{g}} \sum_{j}^{N_{l}} \frac{i^{2}}{j^{2}}*Q\left( i,j \right)$ |
| **long run low gray level emphasis** | $\frac{1}{N_{run}}\sum_{i}^{N_{g}} \sum_{j}^{N_{l}} \frac{j^{2}}{i^{2}}*Q\left( i,j \right)$ | **short run low gray level emphasis** | $\frac{1}{N_{run}}\sum_{i}^{N_{g}} \sum_{j}^{N_{l}} \frac{1}{i^{2}*j^{2}}*Q\left( i,j \right)$ |

**Supplementary Table 2.** Optimization of the model using penalization method.

| **Training set** | Ridge | Elastic net | | Lasso | | |
| --- | --- | --- | --- | --- | --- | --- |
| **Alpha** | 0 | | 0~1 | | 1 |  |
| **Hippocampal model (H+)** | 0.5 | | 0.997 (α = 0.9) | | 0.87 |  |
| **Extrahippocampal model (H-)** | 0.5 | | 0.977 (α = 0.35) | | 0.96 |  |
